# X-ray and computed tomography findings in macrodystrophia lipomatosa of the foot with secondary osteoarthritic changes diagnosed in an elderly female: a case report

**DOI:** 10.1590/0100-3984.2013.0017

**Published:** 2017

**Authors:** Rajesh Sharma, Puneet Gupta, Manik Mahajan, Manjit Arora, Anchal Gupta

**Affiliations:** 1 Department of Radio-diagnosis and Imaging, Acharya Shri Chander College of Medical Sciences Hospital, Sidhra, Jammu, Jammu and Kashmir, India

**Keywords:** Macrodystrophia lipomatosa, Macrodactyly, Computed tomography

## Abstract

Macrodystrophia lipomatosa is a rare entity that is mostly diagnosed in children. It has been very rarely reported in adults. Here, we describe the X-ray and computed tomography findings in a case of macrodystrophia lipomatosa in an elderly female presenting with an enlarged second toe since birth and bony outgrowths causing pressure effects and cosmetic problems.

## Resumo

A macrodistrofia lipomatosa é uma entidade rara, principalmente diagnosticada em crianças, e pouquíssimo relatada em adultos. Descrevemos os achados de radiografia e tomografia computadorizada em um caso de macrodistrofia lipomatosa em uma mulher idosa que apresentava um segundo dedo do pé aumentado desde o nascimento e crescimentos ósseos que causavam efeitos de pressão e problemas cosméticos.

## INTRODUCTION

Macrodystrophia lipomatosa is a rare entity characterized by progressive proliferation of all mesenchymal elements, with a disproportionate increase in fibroadipose tissue. It is a rare cause of congenital macrodactyly and garners clinical attention because of cosmetic concerns, mechanical problems secondary to degenerative joint disease, or the development of neurovascular compression due to large osteophytes. Although its presentation is almost always unilateral, bilateral involvement can occur in rare cases. Macrodystrophia lipomatosa predominantly affects children but can present at any time, from infancy to late adulthood. Here we describe the X-ray and computed tomography (CT) findings in a case of macrodystrophia lipomatosa in an elderly female presenting with an enlarged second toe since birth, together with bony outgrowths, causing pressure effects and cosmetic problems.

## CASE REPORT

**Figure 1:**
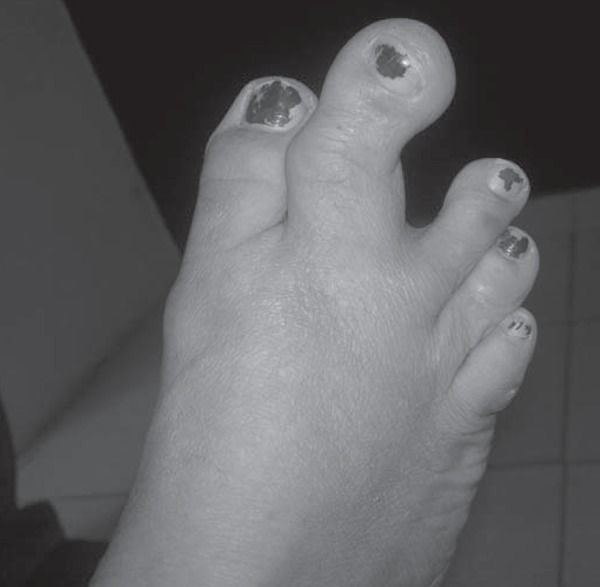
Photograph of the right foot, showing an enlarged second toe and a palpable hard bony lump on its medial aspect.

**Figure 2:**
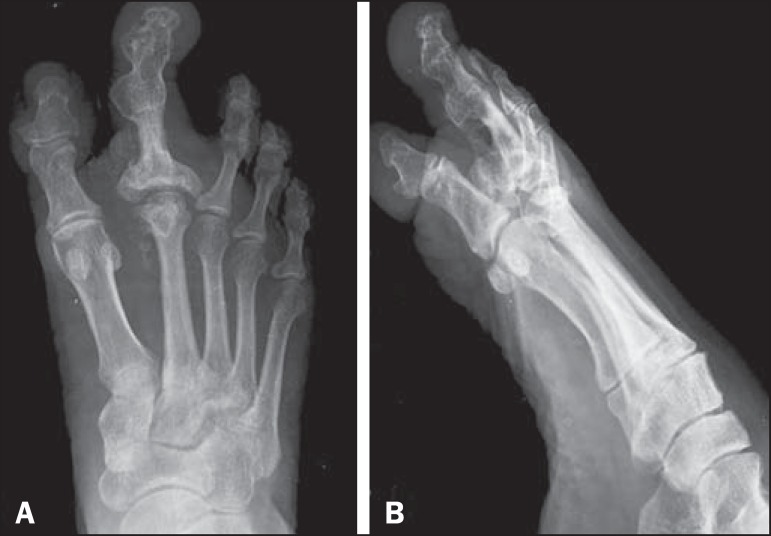
Plain radiographs, in the anteroposterior and oblique planes (**A** and **B**, respectively), of the right foot, showing marked hypertrophy of the second toe with associated enlargement of the soft tissue and phalanges; near complete obliteration of metacarpophalangeal, proximal interphalangeal, and distal interphalangeal joint spaces; and dense bony outgrowths at the medial end of the proximal and distal interphalangeal joints. Note also the thickened, sclerotic cortex of the proximal phalanx of the second toe.

**Figure 3:**
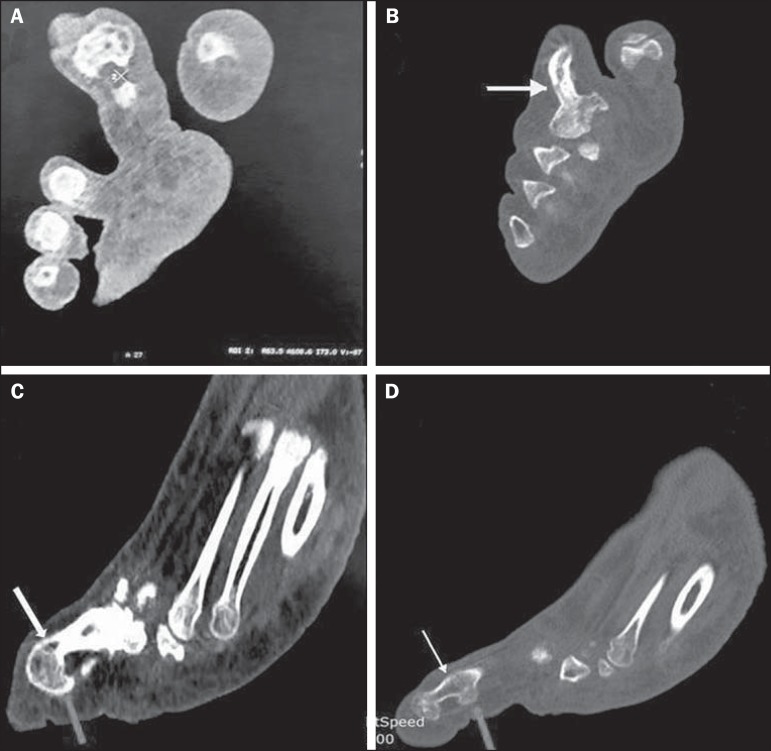
A: Non-contrast-enhanced CT scan, in the axial plane, showing marked soft-tissue hypertrophy containing abnormal hypodense areas of fat attenuation involving the second toe of the right foot. **B:** Non-contrast-enhanced CT scan, in the axial plane, showing the thickened, sclerotic proximal phalanx of the second toe, with an accentuated trabecular pattern (arrow). **C,D**: Non-contrast-enhanced sagittal CT reconstruction, showing fusion of the proximal and distal interphalangeal joints (white arrows), with small bony protuberances arising from the medial end of the joints (gray arrows).

A 55-year-old female presented to an orthopedist complaining primarily of an enlarged second toe, on her right foot, since birth. On inspection and physical examination, a nontender, hard bony lump was palpable on the volar aspect of the enlarged second toe (Figure 1). X-ray and non-contrast-enhanced CT of the foot were ordered for further evaluation. The X-ray showed marked hypertrophy of the right second toe, accompanied by enlargement of the phalanges, soft tissue hypertrophy, degenerative changes (in the proximal and distal interphalangeal joints), and dense bony outgrowths at the medial end of the joints (Figure 2). The CT scan demonstrated marked soft-tissue hypertrophy containing abnormal hypodense (–50 to –120 HU) areas of fat attenuation (Figure 3A). Bony expansion with thickened and sclerotic cortices of the proximal phalanx, fusion of the (proximal and distal) interphalangeal joints, and small bony protuberances arising from medial end of the joints was seen causing compression over the big toe (Figures 3B, 3C, and 3D). The patient was diagnosed with macrodystrophia lipomatosa of the second right toe, together with secondary osteoarthritic changes at the proximal interphalangeal, distal interphalangeal, and metacarpophalangeal joints.

## DISCUSSION

Macrodystrophia lipomatosa is a nonhereditary, congenital developmental anomaly that results in overall progressive overgrowth of all of the mesenchymal elements of a digit, including the digit itself, as well as the phalanges, nerves, and vessels, resulting in gigantism of single digits, multiple digits, or the entire limb^(1,2)^. It typically presents in childhood but can occur in late adulthood or (more rarely) in old age. The overgrowth appears to develop in specific sclerotomes of the body, specifically along the median nerve distribution, in cases of upper limb macrodystrophia lipomatosa, and along the planter nerve distribution, in cases affecting the lower limbs^(3)^. The disease is seen more commonly in the foot than in the hand, with a predilection for the second and third digits^(3,4)^.

The exact etiology of macrodystrophia lipomatosa is not known. Many hypotheses suggesting the cause have been put forth, one of which is that fibrofatty tumors envelop the nerves supplying the enlarged digit, whereas another supposes that macrodystrophia lipomatosa is caused by altered somatic cell formation during development of the limb bud.

Radiological investigations are quite useful in confining the diagnosis. Radiographic evidence of macrodystrophia lipomatosa consists of soft-tissue overgrowth prominently in the median and plantar nerve distributions. Soft-tissue radiolucency is often accompanied by elongated, thickened phalanges with splayed distal ends, resembling a "mushroom" shape^(5,6)^.

Ultrasound reveals large amounts of subcutaneous tissue, infiltration of the muscle, and thickening of the affected nerves. Doppler flow studies show no increased vascularity.

Studies describing the CT features of macrodystrophia lipomatosa are scarce. CT is expected to show overgrowth of the bones, as well as the soft tissues, with a typical distal distribution. The overgrown soft tissue can be identified as normal mature adipose tissue^(7)^.

Magnetic resonance imaging easily demonstrates the excess fibrofatty tissue, which has signal characteristics similar to those of subcutaneous fat: high intensity on T1-weighted sequences, an intermediate intensity on T2-weighted sequences, and low intensity on sequences with fat suppression. In cases of macrodystrophia lipomatosa, the fat is not encapsulated. The fibrous strands within the fatty tissue are seen as low-signal-intensity linear strands on T1-weighted images^(8)^.

Features commonly seen in conjunction with macrodystrophia lipomatosa include osseous protuberances resembling small osteochondromas or osteophytes, degenerative changes in adjacent joints^(9-11)^, and a high incidence of local anomalies such as syndactyly, polydactyly, and (most commonly) clinodactyly.

The differential diagnoses of macrodystrophia lipomatosa are neurofibromatosis type 1 (plexiform neurofibroma), fibrolipomatous hamartoma, lymphangiomatosis, hemangiomatosis with Klippel-Trenaunay-Weber syndrome, Maffucci syndrome, Ollier disease, and Proteus syndrome.

Macrodystrophia lipomatosa patients typically present with two major complaints, cosmetic and mechanical. The cosmetic effects constitute the most common complaint in patients of any age. Mechanical problems are usually seen in adulthood, due to arthritic changes and osseous overgrowth causing compression of neurovascular bundles and resulting in tarsal tunnel syndrome. Surgical intervention is the treatment of choice, depending upon the symptoms and age of the patient, as well as on the extent and severity of the disease.

We conclude that, albeit extremely rare, macrodystrophia lipomatosa can present in elderly patients. Imaging studies, especially CT studies, are helpful in making the definitive diagnosis and can help differentiate macrodystrophia lipomatosa from other causes of localized gigantism, which have different courses, prognoses, complications, and treatments.
